# An adaptive genetic algorithm for selection of blood-based biomarkers for prediction of Alzheimer's disease progression

**DOI:** 10.1186/1471-2105-16-S18-S1

**Published:** 2015-12-09

**Authors:** Luke Vandewater, Vladimir Brusic, William Wilson, Lance Macaulay, Ping Zhang

**Affiliations:** 1 Digital Productivity Flagship, CSIRO, Australia; 2 School of Medicine and Bioinformatics Center, Nazarbayev University, Kazakhstan; 3 Food and Nutrition Flagship, CSIRO, Australia; 4 Menzies Health Institute Queensland, Griffith University, Australia

**Keywords:** adaptive genetic algorithm, logistic regression, Alzheimer's, biomarkers, prediction

## Abstract

**Background:**

Alzheimer's disease is a multifactorial disorder that may be diagnosed earlier using a combination of tests rather than any single test. Search algorithms and optimization techniques in combination with model evaluation techniques have been used previously to perform the selection of suitable feature sets. Previously we successfully applied GA with LR to neuropsychological data contained within the The Australian Imaging, Biomarkers and Lifestyle (AIBL) study of aging, to select cognitive tests for prediction of progression of AD. This research addresses an Adaptive Genetic Algorithm (AGA) in combination with LR for identifying the best biomarker combination for prediction of the progression to AD.

**Results:**

The model has been explored in terms of parameter optimization to predict conversion from healthy stage to AD with high accuracy. Several feature sets were selected - the resulting prediction moddels showed higher area under the ROC values (0.83-0.89). The results has shown consistency with some of the medical research reported in literature.

**Conclusion:**

The AGA has proven useful in selecting the best combination of biomarkers for prediction of AD progression. The algorithm presented here is generic and can be extended to other data sets generated in projects that seek to identify combination of biomarkers or other features that are predictive of disease onset or progression.

## Introduction

Alzheimer's disease (AD), is the major cause of dementia (present in 50-70% of people with dementia). The worldwide number of cases of AD is expected to rise dramatically, from an estimated 35.6 million in 2012 to over 115 million by 2050 [1,2]. Currently there is no effective cure for AD. AD is a progressive and irreversible disease. The growing prevalence, high cost, and an overwhelming impact of AD to the patients, their families, and caregivers increases the urgency of developing treatment that delays the onset and slows the progression of disease.

The identification of risk or benefit factors in pre-clinical AD or in individuals with mild cognitive impairment (MCI) may provide targets for early diagnosis of AD-related dementia. This knowledge, in turn, will help research aimed at prevention or slowing the progression of the disease. Identification of diagnostic features helps targeting of therapeutic trials and appropriate identification of subjects suitable for enrollment into clinical trials. Focusing on suitable diagnostic markers increases chances for success of the trials, and provides the basis for developing population screening tools [3]. The Australian Imaging, Biomarkers and Lifestyle (AIBL) study of aging [4] focuses on periodic collection of feature-rich data from over 1,100 elderly participants classified as healthy controls (HC), MCI or AD. This study includes factors from well-established cognitive tests, emerging biomarker features, and other health and lifestyle factors. This rich dataset enables the use of statistical and machine learning methods for prediction of the onset and progression of AD. It also enables building advanced diagnostic and prognostic tools and contributes to the study of mechanisms of disease development.

The AD is a multifactorial disorder that may be diagnosed earlier using a combination of tests rather than any single test [5]. Because of the diversity of relevant features and their complex relatedness the traditional statistical model building techniques, such as stepwise selection, are limited in their ability to determine high-quality feature sets [6]. Search algorithms and optimization techniques in combination with model evaluation techniques have been used previously to perform the selection of suitable feature sets. Examples include genetic algorithms (GA) [7], LASSO shrinkage [8], particle swarm optimisation and simulated annealing [9]. Logistic regression is commonly used in the field of medical research. The combination of GA with logistic regression (LR) has been proposed for prediction treatment outcome in lung injury [7] and prediction of myocardial infarction in patients with chest pain [10]. It has been shown to perform feature selection significantly better than sequential variable selection methods or random subset selection. Previously we successfully applied GA with LR to neuropsychological data contained within the AIBL study to select cognitive tests important in the progression of AD [11].

Cognitive tests are widely used for diagnosis and predicting the progression of AD as key clinical criteria [5]. Recent research aims to enhance the diagnostic toolkit by adding brain imaging, cerebrospinal fluid (CSF) biomarkers, and plasma biomarkers to provide earlier and more certain diagnostic tools [3,5]. Doecke et al. [6] applied regression models to the AIBL cohort baseline measures data for identification of plasma biomarkers that distinguish HC from AD patients with high sensitivity and specificity. Willette et al [12] identified a group of markers including some cognitive performance measures and structural MR image features for classifying normal cases from MCI or AD patients. They also built a model that predicted the conversion from MCI to AD. Our work focuses on prediction of AD development using advanced machine learning methods. The current report focuses on the development and optimization of an algorithm for feature selection and finding the best combination of biomarkers and demographic features that predict the progression from HC to MCI or AD within 54 months. The dataset used for this study contains 181 biomarker and demographic features presenting a combinatorial solution space of 2181 instances. GA is efficient for searching huge combinatorial spaces and finding combination of variables that can be used for classification problems. Adaptive genetic algorithm (AGA) was shown to improve performance relative to the standard GA when the fitness function is highly epistatic (e.g. the effects of combined genetic mutation are different from their individual effects) [13]. The combination of AGA and LR, is, therefore, suitable for use with large-scale feature selection problem of the complex solution landscape, and was chosen for this study. The algorithm presented here is generic and can be extended to other data sets generated in projects, that seek to identify biomarkers or their combinations that are predictive of disease onset or progression.

## Materials and methodology

**Figure 1:**
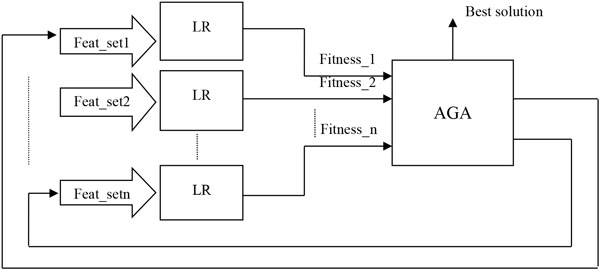
**Diagram of methodology**. Each set of features selected by AGA were fed to LR models, and the output from LR models are used to measure the fitness level of each of the feature combinations. This process is iteratively repeated until the goal solutions are found.

We deployed an AGA for selection of one or more combinations of candidate biomarkers (features) to predict the AD progression with high accuracy. The prediction method deployed the AGA as a search method with a LR algorithm as a fitness function. The overall methodology of the prediction system is depicted in Figure 1. In our algorithm the results from LR with multiple variable sets serve as inputs into the AGA for searching the best combination of features. The features selected by the AGA search are then returned to the LR for iterative identification of the best feature set. A similar method was used earlier in the study of heart disease [7], and for selection of features from cognitive tests that are predictive of AD progression [11].

### Data

We used the data from the Australian Imaging, Biomarkers and Lifestyle (AIBL) study of aging, a prospective longitudinal study of cognition relating to dementia and AD [4]. This collection of data has been collected from more than 1000 participants that are over 60 years old. The AIBL study contained data collected at baseline, 18, 36, and 54 months from the commencement of study. The subset of the data used in this study is from a panel of plasma biomarkers recorded as baseline measures. This panel was used to predict the conversions from HC to MCI/AD within 54 months.

To ensure the completeness of the data we performed data cleaning of biomarker measurements including missing value imputation [6]. The features used in this study included:

• 53 clinical pathology measures (blood analytes)

• 7 measures of circulating metals (plasma analytes)

• 111 protein measures from 151-analyte multiplex panel immunoassay (Human DiscoveryMAP, version 1.0; Myriad RBM, Myriad Genetics, Inc. Austin, Tx)

• 7 plasma measures (plasma levels in pg/mL, including Apolipoprotein E (APOE) levels, Innogenetics and Mehta based ELISAs)

• 3 demographics features: age, sex, and apolipoprotein E allele E4 presence

The details of 181 features are listed in Additional File 1 (Table S1) including the descriptions and ID numbers used throughout the report. The initial cohort included 754 healthy subjects. 170 subjects were excluded from this study: 151 unavailable, 17 deceased, and two that converted to other form of dementia in 54 months. The data set used in this study included 40 HC who converted to either MCI or to AD, and 544 who remained healthy over 54 months. These baseline data were used for building the models for prediction of HC to AD conversion.

### The Algorithm

#### Logistic Regression

Logistic regression is used for probabilistic classification, where a dataset of one or more independent predictors (features) has a dichotomous dependent variable. LR predicts the outcome (typically represented as binary '0' and '1', indicating an absence or presence of a condition) of a set of feature values. The logistic function takes the form (Formula 1):

(1) $$\pi \left(x\right)=1/\left(1+e^{-\left(\beta_{0}+\beta_{1}x_{1}+\ldots +\beta_{m}x_{m}\right)}\right)$$

Where *x_m _*relates to the explanatory variables, *β *is an estimated parameter, and *π*(*x*) is the probability of the dependent variable taking value of '1'. The LR is used to classify each observation of a set of features, and the probabilities used to determine the suitability of the model and, by extension, the feature set. For the AD progression problem, the dependent variable was encoded on a 'conversion' basis - '0' for an HC participant remaining cognitively healthy during the 54 month period and '1' for a participant who converted to MCI or AD within the 54 month period.

#### The Adaptive Genetic Algorithm (AGA)

**Figure 2:**
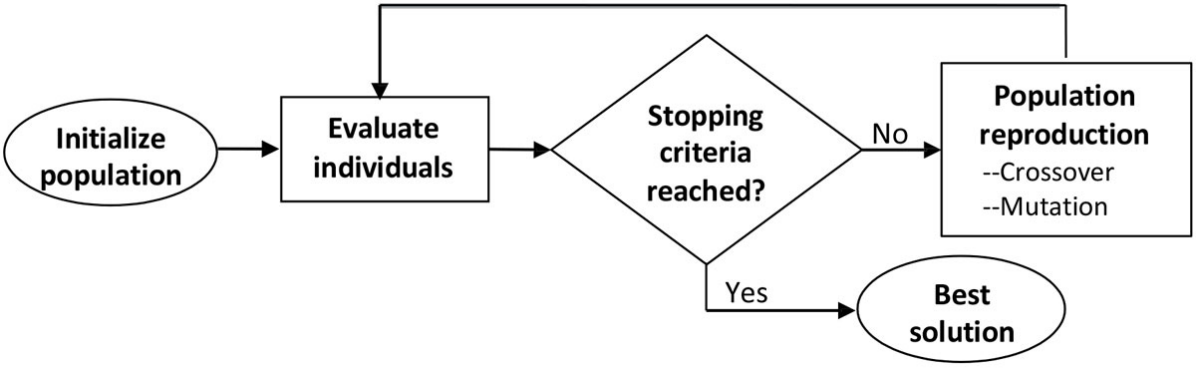
**A diagram representing a typical GA**. Each solution (individual) within a generation is evaluated until the target solution is found. New generation of the population is produced using selection, crossover, and mutation operators that create new solutions. The "best" solution is returned when fitness function reaches target value.

A typical GA, depicted in Figure 2, is an evolutionary process wherein a population of solutions evolves over a sequence of generations. Each individual in the population (called genome or chromosome) represents a candidate solution to the problem. The potential solutions compete and mate with each other to produce increasingly fitter individuals over subsequent generations of solutions. During the reproduction of the next generation, selected individuals are transformed using operations of crossover or mutation under a certain crossover probability *p_c_*, and mutation probability *p_m_*. For feature selection, the initial population is a set of strings (individuals) of length 181 encoding each studied feature by a given position in the strings. The values for each position in each individual was generated randomly using binary encoding (where '1' signifies the inclusion of a feature in the LR). The individuals were assessed for fitness using a cross-validated model. The individuals with highest fitness were passed onto the next generation. The operation was repeated in each generation. The genome with the highest fitness after a number of generations represents the "best" feature set.

The AGA differs from a typical GA in implementation of crossover and mutation operators. In the AGA, the probabilities of crossover and mutation change (adapt) depending on the relative fitness of a solution. This strategy protects high-fitness solutions and disrupts low fitness solutions [13]. The complete disruption of sub-average solutions results in a greater diversity of the solution population and therefore provides for broader exploration of the search space. In addition, it promotes high convergence rates towards globally optimal solutions. In AGA, the probability of crossover is defined by an appropriate function. In this study, the probability of crossover was defined as (Formula 2):

(2) $$p_{c}=\left\{\begin{array}{cc} k_{1}\frac{f_{\text{max}}-f^{\prime }}{f_{\text{max}}-\bar{f}}, & f^{\prime }\geq \bar{f}\\ k_{1}, & f^{\prime }<\bar{f} \end{array}\right)$$

Where *f*_max _is the population maximum fitness, *f*' is the maximum fitness of the two parents of the crossover, $\bar{f}$is the population mean fitness, and *k*_1 _is the adaptive crossover rate (typically 1). We defined the probability of mutation as (Formula 3):

(3) $$p_{m}=\left\{\begin{array}{cc} k_{2}\frac{f_{\text{max}}-f}{f_{\text{max}}-\bar{f}}, & f\geq \bar{f}\\ k_{2}, & f<\bar{f} \end{array}\right)$$

Where *f *is the fitness of the prospective genome to undergo mutation and *k*_2 _is the adaptive mutation rate (usually 0.5).

#### ROC and Fitness Function

To use the LR in a GA, a single number that can be used for 'fitness' judgment is required. In our study, this value was defined by the area under the curve (AUC) of a receiver operating characteristic (ROC) produced by the LR model. The AUC value is a measure of the predictive power of a classifier, where 1 is a perfect prediction, 0.5 is equal to random guessing. For majority of classification systems values of AUC>0.9 indicate excellent predictions, AUC>0.8 are good predictions, while AUC<0.7 indicate poor predictions [14].

The fitness function implements the cross-validated AUC method and returns a fitness value *f *. To facilitate identification of small feature sets that are highly predictive, we introduced a penalty assigned to the larger variable sets in the fitness function. A maximum number of features to be included in the feature set for prediction was defined as a constraint for the function (Formula 4).

(4) $$f=\left\{\begin{array}{cc} 0, & n_{subset}>n_{\text{max}}\\ \bar{AUC}+\rho \times \left(1-\frac{n_{subset}}{n_{total}}\right) & n_{subset}\leq n_{\text{max}} \end{array}\right)$$

$\bar{AUC}$ is the mean AUC value from n-fold cross-validation, *ρ *is an adjustable penalty factor, *n_subset _*is the number of features selected, *n*_max _is a specified maximum feature number, and *n_total _*is the number of features in the dataset. The penalty factor *ρ *pushes the AGA towards selecting a smaller feature subset by improving the apparent fitness of such genomes on a sliding scale. The penalty parameter *n*_max _allows complete rejection of genomes over a specified size, thus reducing the computation involving meaningless genomes. The analysis of this "parameter sweep" can produce insight into effects of feature set size, solution quality, and noise.

#### Model optimization

Model optimization included cross-validation, feature set size optimization, random immigration, and feature sub-setting. Cross-validation was performed to ensure high accuracy of predictions. The feature set size optimization ensured that the sets of selected features are sufficiently small so that they can be interpreted for their biological and clinical meaning. Random immigration feature of the AGA was implemented to prevent convergence towards local minima. Given the large number of potential individual biomarkers (181 features) and their possible combinations, the feature sub-setting was implemented to prevent requirement for excessive computational time.

#### Cross-validation

Repeated n-fold balanced and stratified cross-validation [15] was used in this study to assess the performance of classification models. In 5-fold cross-validation 10 times repeated, the available dataset is split randomly into 5 groups (folds), with the same proportion of each class in each group as the entire dataset. One group is held out as a validation set, while the remaining four groups are combined to form a set on which the regression model is trained. Prediction probabilities are formed with the validation set, and an AUC value is determined. The training-testing procedure is carried out on each fold combination in turn, and a mean of AUC values for five validation groups is taken. The 5-fold cross-validation is repeated 10 times, with the mean of AUC values of each repeat taken to arrive to the final number. Increasing the number of repeats of cross-validation tends to provide better estimate of the accuracy of the method, but it requires more computation time. Therefore, in a GA search, a trade-off needs to be made with a typical choice of 5- to 10-fold validation (depending on dataset size). At the completion of the GA, a Monte-Carlo post-evaluation on the final feature subset of 1000 repeats was made for this study. In the post-evaluation, for each selected feature set, the data were randomly split into 80% for LR model training and 20% for validation. This was repeated for 1000 runs and the AUC was averaged to give the final assessment of accuracy.

## Computational experiments and results

In this study, the LR models were built with 'glm.fit' in the 'stats' R package, using a 'logit' link function [16]. The AGA algorithm was implemented in R whereby we made modifications to the standard GA package code [17]. The crossover and mutation probabilities were calculated for each candidate based on Formulas (2) and (3). They were included in the custom genetic operator functions and supplied to the 'GA' package in R. This modification prevented the re-evaluation of fitness for non-mutated genomes (See Additional File 2 for the code snippets).

The AGA fitness function performed repeated n-fold cross-validation to obtain an averaged AUC value from the validation of a trained LR model. In this study, the AUC values were calculated with the 'HandTill2001' R package [18,19]. By using averaged AUC measures as the fitness values, genetic operations tend to produce a globally optimal feature subset. A variety of model features were utilised to push the AGA towards selection of smaller feature sets, including fitness penalty and size- reduced feature subsets. The well-performing subsets of the overall feature set were empirically defined and provided as the inputs to the AGA.

For search runs, 181 features (Additional File 1: Table S1) were utilized, and the AGA parameters were explored to determine optimized search conditions. Because of their good performance on binary GA problems, a two-point crossover function and a single-bit random mutation function were used. Uniform crossover and two-bit mutation were investigated but they did not provide notable improvements. The initial population creation was random, with a skew of 1/4 to 1/8 '1's in order to bias the algorithm towards selecting smaller feature subsets. A tournament selection operator was used (with n = 2 genomes participating) to promote convergence.

The AGA was executed over 100 'runs' to produce a large number of solution feature sets for a given parameter setup, and the final MC 1000-repeat post-evaluation AUC cross-validation technique was used to compare solutions. Due to the variable quality of cross-validation, the root-mean-square (RMS) error between fitness reported by the AGA runs and the post-evaluation AUC was examined for preliminary runs to select an acceptable number of cross-validation repeats implemented in the fitness function. This was done because more accurate fitness judgment allows the AGA to evolve the best features. Additionally, *ρ, n*_max_, and initial population skew were adjusted to move the AGA quickly toward suitably small feature subsets. The set of parameters common to all results are listed in Table 1.

**Table 1:** Fixed or Default AGA parameters.

Parameter	Value	Notes
Adaptive cross-over rate (*k*_1_)	1	Empirically optimal value [11]
Adaptive mutation rate (*k*_2_)	0.5	Empirically optimal value [11]
Generations	300*	Computation time and solution quality trade-off
Population size	50	Balance between diversity capacity and computation time
Tournament size	2	Low selective pressure

* Unless specified otherwise

The effect of varying feature set size was explored by examination of the maximum values of AUC, while the frequencies of feature selection were checked using histograms. Feature sets were later compared to the stepwise model results to demonstrate the advantages of the AGA approach.

### Cross-validation optimization

**Figure 3:**
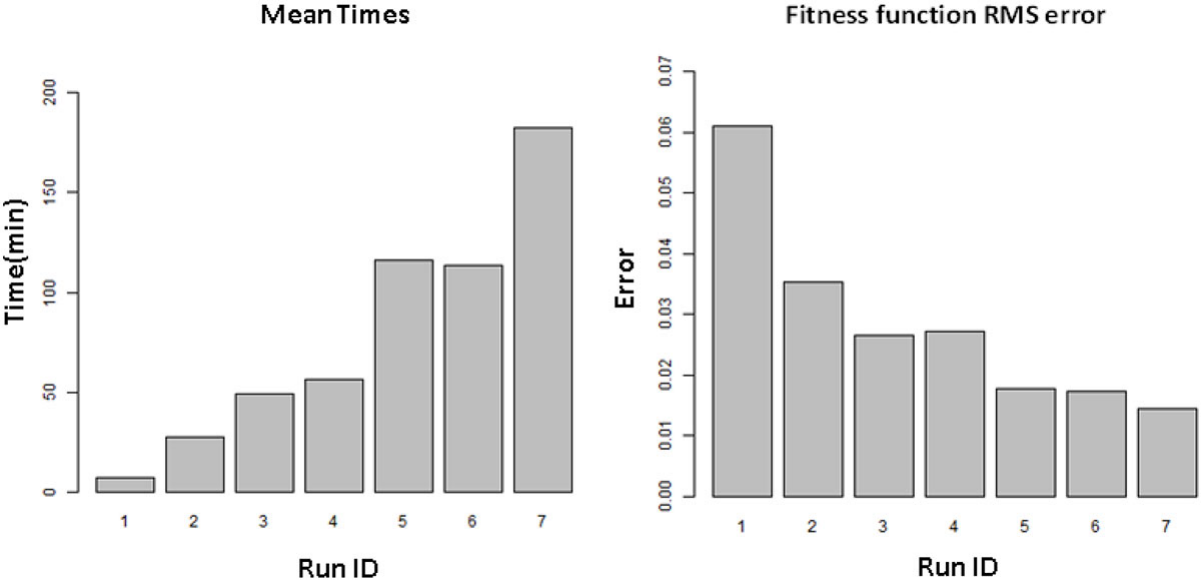
**Cross-validation parameters assessment**. a) Mean time from each set of AGA runs, the AGA was run 100 times with each set of the parameters listed in table 2 respectively, Y axis is the average time of the 100 runs for each run ID. b) RMS errors between the AUC reported by AGA runs (100 for each run ID) and the 1000-repeated post validation (mean of the 1000 AUC). The number of repeats and number of fold for Run ID 1 to 7 are: 1*5, 3*5, 5*5, 3*10,5*10,10*5 and 15*5 respectively. For all these runs, other AGA parameters were: ρ = 0, no penalty to size of feature set; *n_max _*= 50, reduce computational impact; and skew = 1/6, random initial population skewed toward 1/6 features selected.

The number of cross-validation repeats number and the folds count were varied to assess the resulting accuracy of the cross-validated fitness function, quality of overall results, and computational time. Figure 3a depicts the mean AGA run-times approximately as a linear function of the product of cross-validation repeats and folds. Figure 3b shows a decreasing trend in RMS error between the AUC results from the final 1000-repeated post validation and the AGA fitness calculation. To achieve a reasonable balance between computation time and solution quality, we have chosen the parameter set "10-repeated, 5-fold cross-validation" as the "best" to use for our experiments, with "5 repeated, 10 fold cross-validation" parameter set as an alternative choice which produced similar results with slightly increased computation time.

### Feature set size penalty optimisation

Penalty parameter *ρ *was investigated to determine the effects of the size of feature sets. With a coarse parametric sweep (*ρ *= 0-0.6, *n*_max _= 75, skew = 1/6, 10 repeats 5 folds validation), a wide feature set size vs. AUC plot can be elucidated. For experiments, 100 AGA runs for each penalty of 0 to 0.6 with increments of 0.2 were performed (i.e. 100 runs for penalty 0, 0.2, 0.4 and 0.6, respectively). These 400 runs will be named as "full-set runs" in the rest of the paper. The results demonstrated that well-chosen penalty can help select a small number of features. A histogram depiction of feature selection rates in 100 AGA runs for a penalty of 0.6 compared to aggregate feature selection rates across all penalties was supplied in Figure S1 (Additional File 3). It shows similar distributions of the feature selection rates from the runs, with the different sizes of the final selected feature sets. Since smaller sets of features may be more useful in real-life diagnostic applications than larger feature sets, the penalty parameter becomes a useful tool.

**Figure 4:**
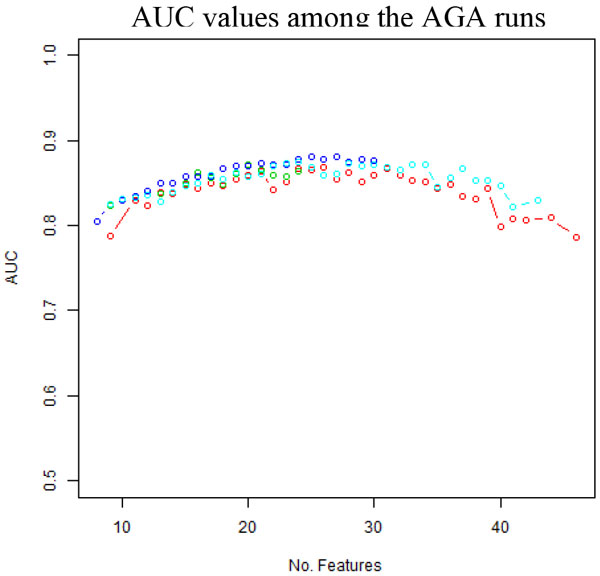
**Comparison of AUC values among the AGA run**. with no subsetting (red), "best half" subsetting (green), "best quartile" subsetting (blue), and random immigrants (cyan), demonstrating that subsetting approach performed the best in terms of the AUC values produced.

The results from the experiments showed a parabolic relationship between the size and classification performance measured by AUC, see Figure 4 (red line created from these experiments) that was created with the results from the feature subsets of size of 9 to 46. The best performing feature set that contained 26 features provided an AUC of 0.87 (Set_26 in Additional File 4: Table S2). A smaller set of 20 features provided an AUC of 0.86 (Set_20 in Additional File 4: Table S2). The feature sets that had sizes greater than 26 or smaller than 20 did not produce better results.

To check the effect of feature set size penalty parameter, we selected a set of 26 features (Set_26.1 in Additional File 4: Table S2) by choosing all features with the frequency greater than 32% in the result from the experiments with *ρ *= 0. The model built from this set of features provided an AUC of 0.85, compared to the 0.87 from the set of 26 features selected by the AGA with a penalty parameter. By selecting the features with the selection frequency over 41%, 20 features were found (Set_20.1 in Additional File 4: Table S2), providing an AUC of 0.84, again worse than the result from the set of 20 features selected by the AGA with a feature size penalty in the fitness function. 30 features were found with a 27% selection frequency threshold (Set_30 in Additional File 4: Table S2) providing an AUC of 0.86, which is lower than the result produced by a smaller feature set selected by AGA with a penalty parameter. These results have shown that AGA is sensitive to the effects of the combination of the features, and the penalty parameter is useful in model fitness evaluation.

### Random immigration performance

To lessen population stagnation and increase diversity, a random immigration feature in the AGA was implemented. The 'age' (measured in generations number) of each genome was tracked, and any modifications to an individual in crossover or mutation would result in a reset of its age. After reaching a pre-defined 'lifespan' (maximum age), the genome was removed from the population and replaced by a new randomly-created genome. The lifespan implemented was 30 generations providing genomes with sufficient time to produce offspring. A protection feature was implemented as a form of elitism, allowing the highest fitness genome to age beyond its lifespan indefinitely (if there were multiple genomes with the same best fitness, 50% were protected).

**Figure 5:**
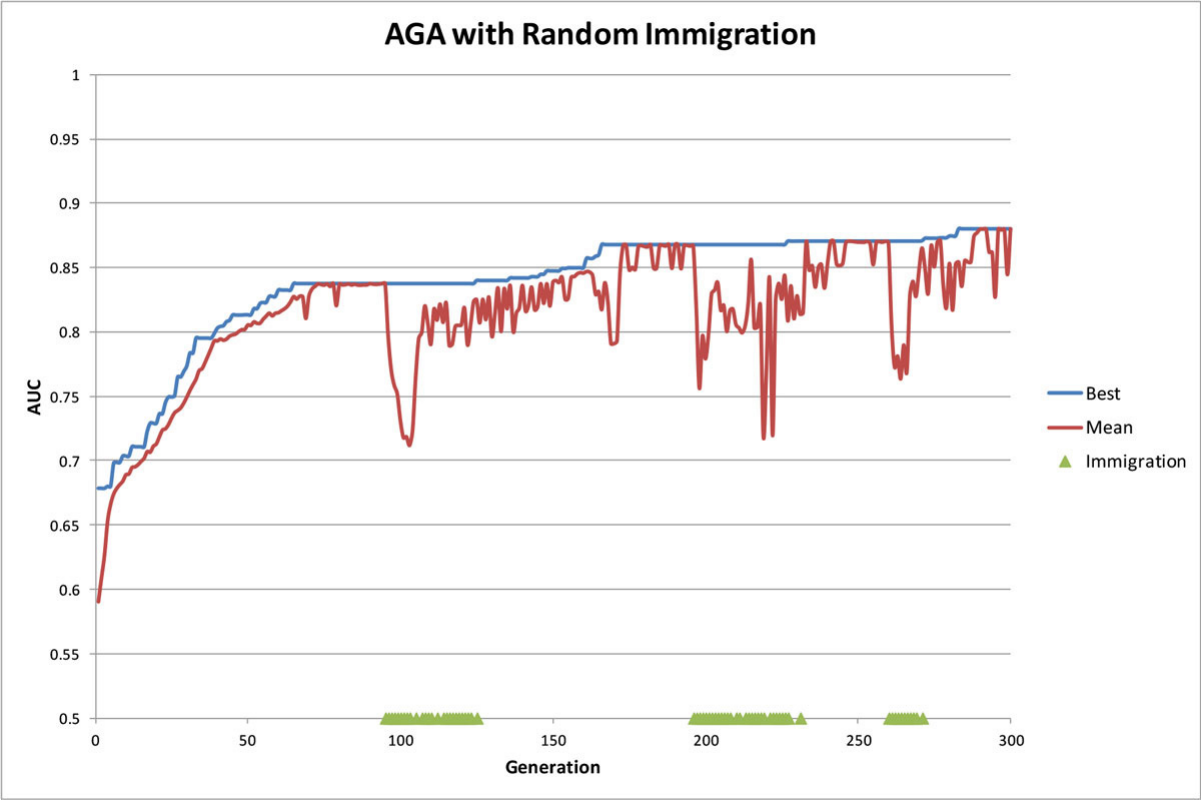
**An AGA run with random immigration (lifespan of 30) demonstrating the convergence properties**.

Figure 5 shows a typical example of the AGA with random immigration run - the current best genome (blue), population mean AUC (red), and points of immigrant influx (green triangles). The early convergence patterns typical of a GA were visible until approximately generation 64, after which the population lost diversity and stagnated. At this point, adaptive crossover and mutation rates were mildly effective at creating diversity in the population (seen as the small 'dips'); however the selective pressure of tournament selection negated the impact. The first influx of random immigrants over generations 95-125 triggered a large amount of diversity in the population, powering the adaptive crossover and mutation to lead to an increase in the best solution. This process repeated until the termination at 300 generations. We speculate that longer computation may provide better results but the convergence pattern shows that future gains are likely to be small.

The experiments results comparing the AGA with immigration and without immigration are shown in Table 2. The AUC values were from the 400 AGA runs (named as "full set runs", 100 runs for each of the penalty values 0, 0.2, 0.4 and 0.6). Given the convergence properties of the immigrants trials (Figure 5), it is feasible that running the AGA with immigrants for more generations may provide better results. Our experimental result (Table 2) indicated that added generations provides the AGA with more scope to improve the solutions, however, the trade-off is computation time. We can see that only slightly improved results come with the doubling of computation time.

**Table 2:** Comparison of statistics for AGA runs with immigration and without immigration.

Run ID	Generation	Lifespan	Immigration	Max AUC	Median AUC	Lower Quartile	Upper Quartile	Mean Time (minutes)
1	300	300	No	0.87	0.82	0.81	0.84	116

2	300	30	Yes	0.88	0.84	0.83	0.85	226

3	600	30	Yes	0.89	0.86	0.85	0.87	436

Other parameters used in the AGA:*ρ *= 0 - 0.6, in 0.2 steps; *n*_max _= 50; Skew = 1/6; 10 repeats and 5 fold cross validation.

### Sub-setting features for AGA by selection frequency approach

While the penalty parameter *ρ *is able to push the AGA selection toward smaller feature sets, a more intuitive approach of feature reduction is providing the AGA with only a selected set of features. This selection feature subset was chosen from the most commonly selected half (90 total) of the features in the full sweep result from the AGA runs with no immigrants and penalty parameter *ρ *value from 0 to 0.6 ("full-set runs" as shown in Section "Feature set size penalty optimization"). This subset of the features will be stated as "best half" in the rest of the paper. A 'top quartile' subset was taken by choosing the top half of the previous runs (45 total features), named as "best quartile" later. The subsets were then used for the new AGA runs swept through penalties *ρ *of 0, 0.1, and 0.2. The results were compared with previous results (see Figure 4).

The "best quartile" approach chose a best model of 30 features (Set_30.1 in Table S2, Additional File 4) with an AUC of 0.89. The random immigrant strategy let the AGA choose a few bigger feature sets that produced the equivalent AUC values, however the computation time was much higher.

### Sub-setting features for AGA by best model combination approach

The approach of feature reduction by selection rate from the full set runs introduced to the new AGA has been shown to work well in accuracy of predictions, however it may lose features that are important in combination effects. To mitigate this, the sub-setting was performed by selecting features that were present in the top 5% of models by AUC from "full-set runs" (named as "top 5% by AUC"), in addition to the "best half" by selection frequency. This feature set contains 126 out of the full 181 features. Our experimental result did not show an appreciable difference with the sub-setting runs with the "best half" and "best quarter" feature sets (Additional File 5: Figure S2).

### Overall model performance and experimental results

Combining the results from the experiments with penalty sweeps, immigration trials, and sub- setting approaches introduced to the AGA, the overall comparison of the prediction models from the feature set with different sizes demonstrated that the optimal or near optimal feature set sizes were between 22 and 34 features (Table 3). The feature sets with a length of 30 features built the prediction models that produced the highest AUC of 0.89. The corresponding parameters used in the AGA that selected feature sets are also listed the in Table 4. The corresponding features included in the feature sets selected by the AGA can be found in Additional File 4.

The results from the AGA were compared with those from randomly generated models and from stepwise algorithm selected models. One hundred different feature sets per size were chosen at random, with a 100-repeated 5-fold AUC value taken, and a stepwise model ('stepAIC' in the MASS package [20]) was paused at specific steps to calculate the 1000-repeated 5-fold AUC value. The result (Table 3) showed significant difference between the models selected by AGA and stepwise or randomly chosen (p < 0.001).

**Table 3:** Best performing models for feature set sizes ranging from 10 to 38.

AGA Run Parameters and Result								Random	Stepwise
**#**	**Size**	**Rho**	**Subset**	**Immi- grants**	**Generations**	Feature set**	**AUC**	**AUC**	**AUC**

**1**	10	0.6	None	30	300	Set_10	0.83	0.59	0.76

**2**	14	0.1*	Quartile	N/A	300	Set_14	0.85	0.60	0.78

**3**	18	0.1*	Quartile	N/A	300	Set_18	0.86	0.59	0.79

**4**	22	0.1*	Quartile	N/A	300	Set_22	0.87	0.59	0.80

**5**	26	0*	Quartile	N/A	300	Set_26	0.88	0.60	0.81

**6**	30	0*	Quartile	N/A	300	Set_30	0.89	0.59	0.82

**7**	34	0.2	None	30	600	Set_34	0.88	0.59	0.82

**8**	38	0	None	30	300	Set_38	0.85	0.60	0.82

* Penalty applied to the AGA started with only "best quartile" subset.

** Included features can be found in Additional File 4: Table S2.

The results were compared with models from the randomly selected feature sets (same sizes) and stepwise selected models

**Table 4:** Logistic regression model built with the selected 10 features.

Variable #	21	83	106	111	117	123	140	172	177	180
Coefficient	-0.56	1.03	-1.73	-1.75	3.57	-9.76	1.84	-3.20	-1.18	0.09

P_value	0.086	0.046	0.025	0.014	0.003	0.007	<0.001	0.001	0.015	0.002

Variable details are included in Additional File 1: Table S1.

To assess the contribution of each variable to the prediction accuracy, we built models using the whole set of data and the selected variable sets. Table 4 shows the coefficient and the p_value of each variable in the logistic regression model built with the set of 10 features (#1 in Table 3). The result indicated that the individuals who have higher values of variables 83, 117, 140, and 180 (RBM_CEA, RBM_IL-13, RBM_Myoglobin and Age), and lower values of variables 21, 106, 111, 123, 172 and 177 (Pathology_K, RBM_HB-EGF, RBM_IgA, RBM_IL-3, Plasma_ApoE and Plasma_Mehta_AB42) have higher risk of getting into MCI/AD within 54 months. For more information about the variables, see Additional File 1 Table S1

## Conclusion and discussion

An AGA with LR approach has been explored in terms of parameter optimization, to find the best biomarker combinations for prediction of AD progression with high accuracy. The conversion of HC to MCI or AD in 54 months was predicted well by the AGA with LR approach, with a best model of 30 features producing a cross-validated AUC of 0.89. The feature sets with smaller sizes that produced high AUC values had big overlaps in composition, but were not the subsets of the bigger sets. For example, a smaller feature set of 22 features that produced result with AUC = 0.87, excluded 12 features from the 30 feature set, while it added additional four features, showing the combination selective ability of the AGA. Additionally, the selection of features purely by selection frequency >27% from the full set runs, as highlighted earlier, produced a set of 30 features giving an AUC of 0.86, suggesting that the AGA for selection of the combination effects improve the predictive power of the model.

The best models were produced by the feature sets with sizes between 22 and 34, where smaller sets have slightly lower predictive accuracy, and larger sets increase the model noise. These factors clearly demonstrated the power of the AGA approach for feature selection that apply to modelling of prediction of AD progression. The found solutions were not sensitive to noise due to set size.

The selection of best quartile subsets applied to the AGA worked well with this particular set of features, indicating that we can run the AGA with the full set of features in less generations and then use the subset selection approach to reduce computation time without compromise the quality of models. While it may be argued that the sub-setting approach may reduce the power of the AGA to determine quality feature combinations from a whole set, we found that the results based on selection of the top 5% features did not produce better results. This also might be related to the particular set of features that we used in this study. For example most of the biomarkers might have independent biological functions. The random immigration approach with a lifespan of 30 generations was able to better explore the larger search space on non-subset problems to preserve the best solutions. Providing AGA with more generations before termination increased result quality, however computation time was far too large to prefer this method to the sub-setting.

The combined AGA was applied to feature selection, and we showed that it is useful in selecting the best combination of biomarkers for prediction of AD progression. The selected features were compared with the literature reports, and we found that our highly selected biomarkers, for example blood levels of folate and vitamin B12, were reported to be associated with AD [21,22]. A few biomarkers that associated with immune system and Cholesterol metabolism (e.g. IL-13, IL-3, CRP, I-309, IgA, IgE, PARC and Lipoprotein(a) ) were also very frequently selected by our algorithm. Our results are consistent with some recent research reports [23-25]. Our algorithm identified a possible role of Myoglobin (a single-chain globular protein) and CEA (a glycoprotein involved in cell adhesion) in AD progression prediction as part of the combination of the feature sets. APoE_ECU that is total APoE and Mehta_AB42, Plasma AB42 levels in pg/mL (MEHTA), also appeared as possible important plasma biomarkers as part of the combined feature sets. These results encourage us for further analysis of the feature sets to determine the biological context and to find the smallest set of biomarkers for prediction of AD progression. To integrate the biological interpretation into the algorithm and understand the biological meaningful application will be the next step of the work.

The developed model and the general model optimization methods presented here can be used for studies of other diseases. Further work may also involve applying the algorithm for prediction of the multi-classes of the progression (e.g., converted at the different time points) and prediction of the rate/speed of progression. Additionally, investigation into over-fitting issues within the AIBL dataset should be made (by use of another dataset to validate for example ADNI). We expect that the cross- validation method employed here will preserve the generality of the results.

## Competing interests

Authors have no competing interests relevant to this paper.

## Authors' contributions

LW wrote the code and performed initial data analyses. VB designed data analysis, and participated in the design of methodology. WW and LM contributed to the study design and oversaw the project progress. PZ designed the methodology and data analysis, performed data analysis and supervised the project. LW, PZ, and VB wrote the paper. All authors read the paper, made comments, and agreed with the content.

## Supplementary Material

Additional file 1 **Table S1: Whole set of the features used for this research**. This excel file includes 5 tables displayed in 5 seperate sheets. Table S1.1 lists the pathology features used in this research. Table S1.2 is the list of Metals features. Table S1.3 is the set of protein measures from 151-analyte multiplex panel immunoassay (RBM features). Table S1.4 a nd Table S1.5 list the plasma features and demographic feature used for this researh respectively. Click here for file

Additional file 2 **R code shows the modification of "GA" package**. Click here for file

Additional file 3 **Figure S1: Histograms demonstrating feature selection frequencies from the AGA runs**. The feature selection frequencies are from 100 AGA runs at 0.6 penalty versus 400 AGA runs with penalties 0-0.6 (100 runs for each of the penalties 0, 0.2, 0.4,0.6 ). It shows similar distributions of the feature selection rates from the runs, with the different sizes of the final selected feature sets. Click here for file

Additional file 4 **Table S2: Feature sets selected by Genetic Algorithm from the experiments corresponding to the description in the paper**. Click here for file

Additional file 5 **Figure S2: Comparison of AUC with best 5%, best half and best quartile subsetting**. Click here for file

## Declaration

The publication charges were funded by CSIRO, Australia.

This article has been published as part of *BMC Bioinformatics *Volume 16 Supplement 18, 2015: Joint 26th Genome Informatics Workshop and 14th International Conference on Bioinformatics: Bioinformatics. The full contents of the supplement are available online at http://www.biomedcentral.com/bmcbioinformatics/supplements/16/S18.
