# Supplementary material for: An adaptive genetic algorithm for selection of blood-based biomarkers for prediction of Alzheimer's disease progression
Source: BMC Bioinformatics. 2015 Dec 9;16(Suppl 18):S1. doi: 10.1186/1471-2105-16-S18-S1 (PMC4682419; doi:10.1186/1471-2105-16-S18-S1)
Supplement: Additional file 2 — R code shows the modification of "GA" package. [file 1471-2105-16-S18-S1-S2.pdf]

R Code

'GA' Package Modification

File: R\ga.R

Lines: 248-259

GA version 2.0 code:

```
# mutation
if(is.function(mutation) & pmutation > 0)
{ for(i in 1:popSize)
  { if(pmutation > runif(1))
    { Mutation <- mutation(object, i)
      Pop[i,] <- Mutation
      Fitness[i] <- NA
    }
  }
  object@population <- Pop
  object@fitness <- Fitness
}
```

Modified to:

```
#mutation – optionally return genome and fitness in a list to facilitate AGA
if(is.function(mutation) & pmutation > 0)
{ for(i in 1:popSize)
  { if(pmutation > runif(1))
    { Mutation <- mutation(object, i)
      if(is.list(Mutation)) {
        Pop[i,] <- Mutation$genome
        Fitness[i] <- Mutation$fitness
      } else {
        Pop[i,] <- Mutation
        Fitness[i] <- NA
      }
    }
  }
  object@population <- Pop
  object@fitness <- Fitness
}
```
