# Supplementary material for: An adaptive genetic algorithm for selection of blood-based biomarkers for prediction of Alzheimer's disease progression
Source: BMC Bioinformatics. 2015 Dec 9;16(Suppl 18):S1. doi: 10.1186/1471-2105-16-S18-S1 (PMC4682419; doi:10.1186/1471-2105-16-S18-S1)
Supplement: Additional file 3 — Figure S1: Histograms demonstrating feature selection frequencies from the AGA runs. The feature selection frequencies are from 100 AGA runs at 0.6 penalty versus 400 AGA runs with penalties 0-0.6 (100 runs for each of the penalties 0, 0.2, 0.4,0.6 ). It shows similar distributions of the feature selection rates from the runs, with the different sizes of the final selected feature sets. [file 1471-2105-16-S18-S1-S3.pdf]

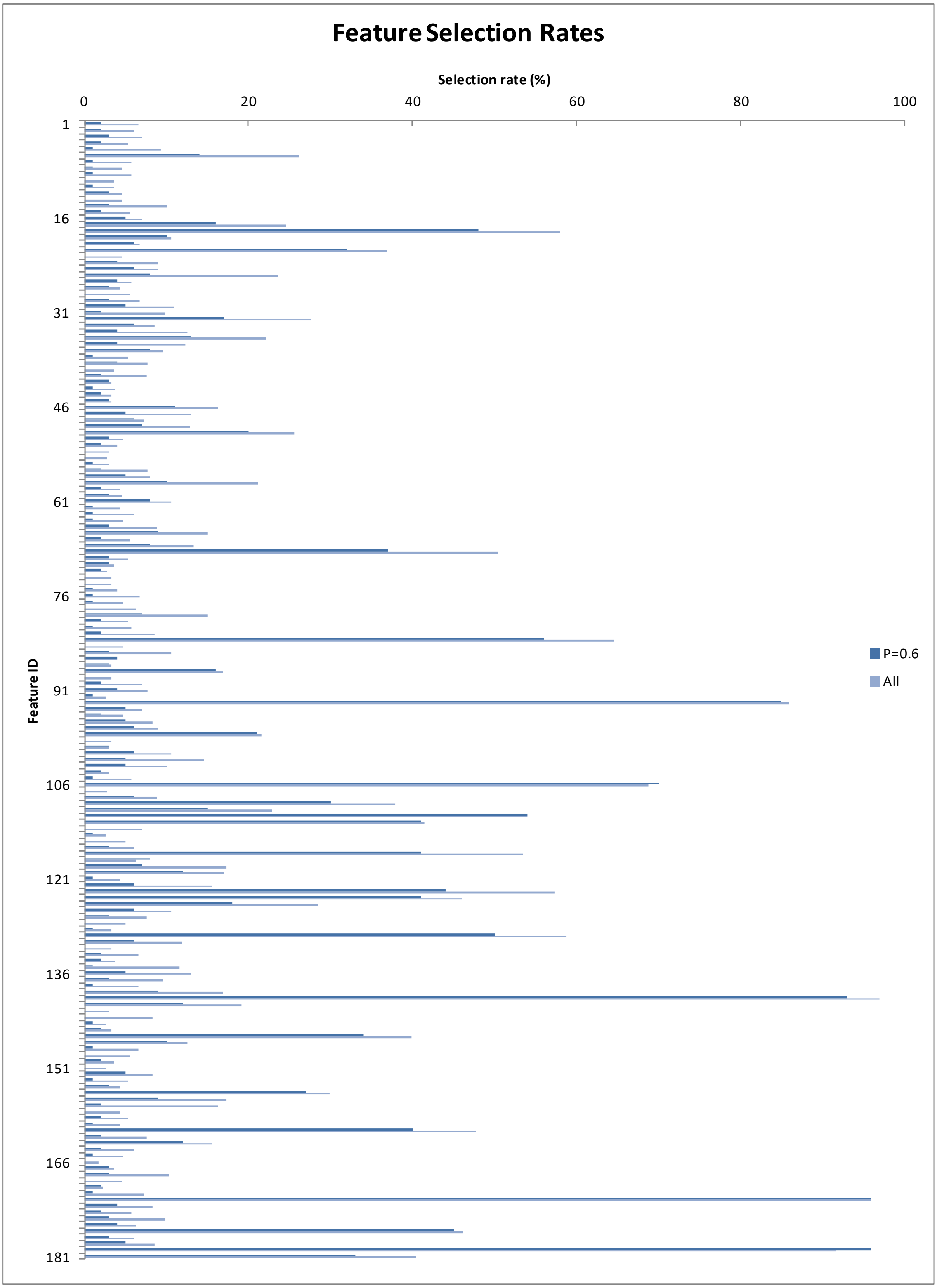

Figure S1: Histograms demonstrating feature selection frequencies from the AGA runs from 100 AGA runs at 0.6 penalty versus 400 AGA runs with penalties 0-0.6 (100 runs for each of the penalties 0, 0.2, 0.4,0.6). It shows similar distributions of the feature selection rates from the runs, with the different sizes of the final selected feature sets.
