# Supplementary material for: An adaptive genetic algorithm for selection of blood-based biomarkers for prediction of Alzheimer's disease progression
Source: BMC Bioinformatics. 2015 Dec 9;16(Suppl 18):S1. doi: 10.1186/1471-2105-16-S18-S1 (PMC4682419; doi:10.1186/1471-2105-16-S18-S1)
Supplement: Additional file 5 — Figure S2: Comparison of AUC with best 5%, best half and best quartile subsetting. [file 1471-2105-16-S18-S1-S5.pdf]

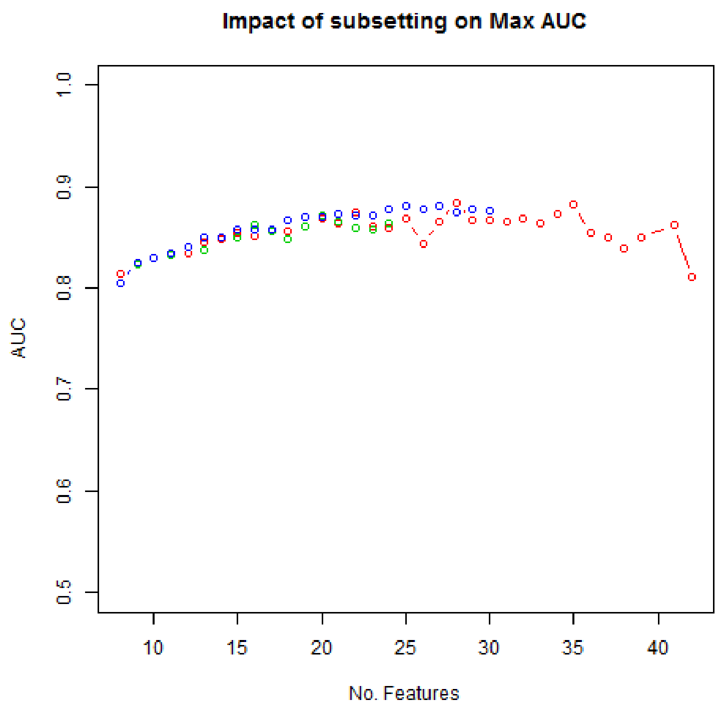

Figure S2: Comparison of AUC with best 5% (red), and previous best half (green) and best quartile (blue) subsetting
